# Sustainable [EMIM]OAc–Nanocellulose Ionogels for Flexible High-Performance Supercapacitors

**DOI:** 10.1021/acsomega.6c05240

**Published:** 2026-08-14

**Authors:** Miguel Morales, Vicente Roda, Attila Husar

**Affiliations:** † CIEFMA-Department of Materials Science and Engineering, Universitat Politècnica de Catalunya, Barcelona-Tech, Campus Diagonal Besòs-EEBE, Barcelona 08019, Spain; ‡ Centre for Research in Multiscale Science and Engineering of Barcelona, Universitat Politècnica de Catalunya, Barcelona-Tech, Campus Diagonal Besòs-EEBE, Barcelona 08019, Spain; § GReCEF-Department of Fluid Mechanics, Universitat Politècnica de Catalunya, Barcelona-Tech, Campus Diagonal Bess-EEBE, Barcelona 08019, Spain

## Abstract

The increasing global demand for sustainable energy storage systems has intensified the investigation for advanced materials that combine high performance with environmental compatibility. In this context, ionogel electrolytes (IGEs) have emerged as promising candidates for all-solid-state energy storage devices owing to their outstanding thermal and chemical stability. Nevertheless, simultaneously achieving high ionic conductivity, mechanical flexibility, and thermal robustness remains a major challenge for conventional ionic liquid-based polymer ionogels. This work presents the fabrication of a supercapacitor based on an eco-friendly ionogel electrolyte composed of 1-ethyl-3-methylimidazolium acetate ([EMIM]­OAc) and a carboxymethyl cellulose nanofibrillar network (CMCNF) processed from carboxymethyl cellulose (CMC) using a combined physicochemical and mechanical fibrillation process. Flexible electrodes were prepared via a water-based slurry method using active carbon, acetylene black and polyvinylidene fluoride (PVDF). Material characterization revealed a homogeneous ionogel structure with strong intermolecular interactions and an amorphous nature, facilitating efficient ion transport. The supercapacitor achieved a specific capacitance of 144 F g^–1^ at 1 A g^–1^, while operating over an expanded voltage window about 2.0 V and exhibiting good thermal stability up to 250 °C, thereby overcoming the limitations of conventional aqueous electrolytes. Furthermore, the device exhibited remarkable mechanical flexibility and long-term durability, retaining about 80% of its initial capacitance after 10,000 charge–discharge cycles. These findings demonstrate that the proposed nanocellulose-based ionogel provides a sustainable and scalable strategy for high-performance flexible supercapacitors, effectively bridging the performance gap between high-power aqueous systems and high-energy organic electrolyte devices.

## Graphical abstract

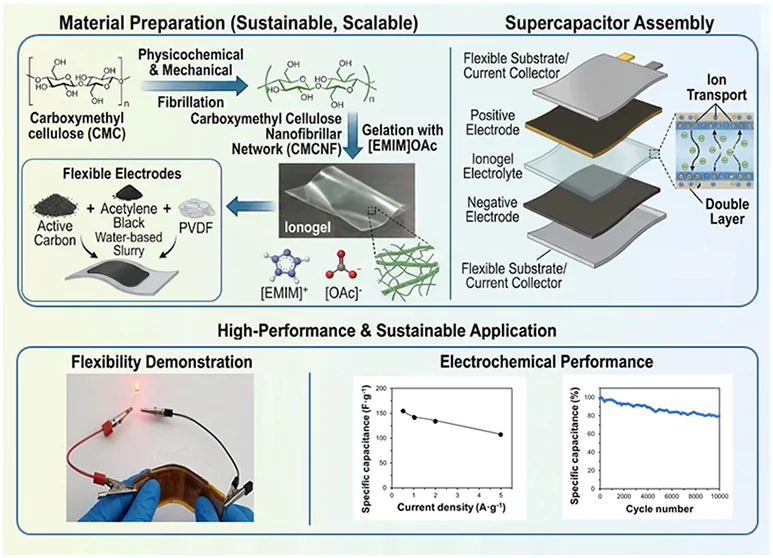

## 1 Introduction

The global surge in energy consumption, together with the urgent need for industrial decarbonization and the transition toward green electronics and wearable technologies, has intensified the demand for advanced energy storage systems (ESS) that combine high performance, mechanical resilience, and environmental sustainability.^1–4^ While lithium-ion batteries (LIBs) currently dominate the market, their intrinsic limitations in power density, safety, and cycle life have accelerated interest in alternative technologies.^5–7^ Among these, supercapacitors (SCs) have emerged as promising candidates due to their rapid charge–discharge capability, high power density, and outstanding cycling stability.^8,9^ Their energy storage relies on electrical double-layer capacitance (EDLC) and/or pseudocapacitance, enabling them to bridge the gap between conventional capacitors and batteries for high-power and long-life applications.^10–15^ In EDLCs, energy is stored through electrostatic ion adsorption at the electrode/electrolyte interface, while pseudocapacitive systems involve faradaic charge transfer processes with fast kinetics, enabling higher capacitance values.^10,11,16^ Consequently, SCs combine high capacitance, power density, long cycle life, and rapid charge–discharge capability, making them well suited for flexible, wearable, and miniaturized electronics.^10,16–18^ Quasi-solid-state electrolytes have therefore attracted considerable attention because they combine the high ionic conductivity of liquid electrolytes with the mechanical integrity and safety of solid materials.^19^ Their ionic conductivity (1–10 mS·cm^–1^) approaches that of liquid electrolytes,^20,21^ while enabling flexible device architectures with improved electrochemical stability and reduced leakage risks.^22,23^

Electrolytes for ESSs are generally classified into liquid electrolytes, solid-state electrolytes (SSEs), and gel polymer electrolytes (GPEs). Among them, GPEs are particularly attractive because their three-dimensional polymer network confines the liquid electrolyte, ensuring efficient ion transport and dimensional stability.^24^ Ionic liquids (ILs), salts with melting points below 100 °C, are widely employed in GPEs owing to their wide electrochemical stability window, high ionic conductivity, negligible vapor pressure, and nonflammability.^25^ These properties enable high-voltage supercapacitors with enhanced energy density, while recent developments in task-specific and bioderived ILs have further improved their sustainability.^26^ Ionogels, in which ionic liquids are immobilized within polymeric or inorganic networks, combine high ionic conductivity with excellent mechanical flexibility and thermal stability.^27^ Their nonvolatile nature minimizes leakage and evaporation, making them particularly attractive for flexible energy-storage devices.^28^ Recent advances have demonstrated highly stretchable, tough, and even self-healing ionogels with enhanced ion transport and excellent capacitance retention under repeated deformation.^23,29–31^ Consequently, ionic conductive gels have become promising materials for wearable electronics, artificial intelligence systems, flexible displays, and foldable energy-storage devices.^32–38^ More recently, advanced molecular and structural design strategies have enabled ionogels with simultaneously enhanced ionic conductivity, mechanical robustness, and electrochemical stability, further demonstrating their potential as next-generation electrolytes for flexible energy-storage devices.^39,40^ Their performance strongly depends on the polymer matrix.^41^ Although synthetic polymers remain the preferred choice because of their excellent mechanical properties and biocompatibility,^42,43^ their dependence on nonrenewable petrochemical resources raises important environmental concerns.^44,45^

In light of the growing demand for sustainable energy storage systems, considerable efforts have focused on developing green electrolytes and polymer matrices. Renewable cellulose has emerged as a promising candidate because of its abundance, biodegradability, and environmental compatibility.^46–48^ Among cellulose-based materials, nanocellulose (NC) has attracted particular attention owing to its tunable surface chemistry, excellent mechanical properties, and ability to form interconnected networks that promote ion transport.^8,24^ Its abundant hydroxyl groups also facilitate functionalization with conductive polymers, redox-active species, and inorganic nanofillers, leading to enhanced electrochemical performance in hybrid systems.^49^ In parallel, various cellulose-based conductive gels have been developed by combining cellulose with conductive media such as inorganic salts,^44^ ionic liquids (ILs),^45^ or conducting polymers.^50^ In particular, cellulose-based ionogels containing ILs combine high ionic conductivity with excellent low-temperature performance, making them promising sustainable electrolytes for flexible electronics.^34,45,51^

Despite these advantages, cellulose processing remains challenging because its strong hydrogen-bonding network and high crystallinity limit its solubility in conventional solvents.^52^ Imidazolium-based ionic liquids (ILs), including 1-ethyl-3-methylimidazolium acetate ([EMIM]­OAc), 1-butyl-3-methylimidazolium chloride ([BMIM]­Cl), and 1-allyl-3-methylimidazolium chloride ([AMIM]­Cl), are effective cellulose solvents that disrupt intermolecular hydrogen bonding, producing homogeneous solutions with tunable physicochemical properties.^52–55^ Their dissolution ability arises from interactions between IL anions and cellulose hydroxyl groups, while cations stabilize the solvent structure, enabling the fabrication of advanced cellulose-based ionogels and composite electrolytes.^8,24,56–59^ In this context, cellulose-based ionogels have emerged as sustainable alternatives to conventional fluorinated polymer electrolytes. Nanocellulose-based and methyl cellulose/ionic liquid ionogels have demonstrated promising ionic conductivity (0.32–0.94 mS·cm^–1^) and specific capacitances up to 160 F·g^–1^, along with reduced equivalent series resistance (10.2 Ω·cm^2^),^60^ and cycling stability (∼96% retention after 20,000 cycles),^25^ confirming their suitability for flexible solid-state supercapacitors.^61^ More recent strategies have focused on hybrid nanocellulose systems incorporating conductive and pseudocapacitive materials. Cellulose nanofiber (CNF) aerogels combined with carbon nanotubes (CNTs) and MnO_2_ have improved capacitance and cycling stability,^49^ while hierarchical CNF/graphene and CNF/MoS_2_ composites have enhanced rate capability through their porous architectures.^62^ Likewise, nanocellulose/polymer ionogels containing ionic liquids have achieved high ionic conductivity together with excellent flexibility and mechanical robustness.^29,63^ However, several challenges still hinder the practical implementation of nanocellulose-based ionogels in sustainable supercapacitors. Their ionic conductivity remains lower than that of liquid electrolytes because of restricted ion mobility within hydrogen-bonded networks,^26,64^ while balancing mechanical strength with efficient ion transport and achieving good electrode/electrolyte interfacial compatibility remain difficult.^65–68^ Furthermore, although conductive nanomaterials improve electrochemical performance, they often compromise device sustainability, and issues related to scalability, structural control, and long-term stability are still unresolved.^26,65,67^

To address these limitations, this work adopts a rational materials design based on a carboxymethyl cellulose nanofibrillar network (CMCNF) combined with the low-volatility ionic liquid 1-ethyl-3-methylimidazolium acetate ([EMIM]­OAc). Carboxymethyl cellulose (CMC) was selected as the precursor due to its hydroxyl and carboxyl functional groups, which enable the formation of a stable nanofibrillar network and provide effective interactions with the ionic liquid matrix. The resulting CMCNF architecture provides a mechanically robust scaffold while maintaining the ionic liquid as the continuous phase for ion transport. This design enables the preparation of homogeneous ionogel electrolytes with enhanced mechanical stability and efficient ionic conduction. The resulting CMCNF-based ionogel enables the fabrication of a sustainable solid-state supercapacitor operating over a 2.0 V voltage window with thermal stability up to 250 °C, providing a scalable strategy that bridges the gap between high-power aqueous supercapacitors and high-energy organic electrolyte systems.

## 2 Experimental Procedure

### 2.1 Fabrication of [EMIM]­OAc–CMCNF Ionogel

To fabricate the precursor of carboxymethyl cellulose nanofibrillar network (CMCNF), CMC was first dispersed in deionized water at a concentration of 3 wt % and magnetically stirred at 50 °C until a homogeneous solution was obtained. Lowering the pH partially protonates the carboxylate groups of CMC, thereby reducing electrostatic repulsion between polymer chains and promoting their association through intermolecular hydrogen bonding. This process induces the formation of localized fibrillar supramolecular aggregates within the aqueous medium. The resulting dispersion was subsequently subjected to probe ultrasonication mechanically disintegrate these aggregates into a stable nanofibrillar dispersion while preserving the self-assembled fibrillar structure. The obtained material was denoted as carboxymethyl cellulose nanofibrillar network (CMCNF). The CMCNF dispersion was purified by repeated centrifugation and redispersion cycles using deionized water until a neutral pH was achieved. Afterward, the CMCNF suspension was concentrated to a solid content of 1 wt % for subsequent use. Prior to use, [EMIM]­OAc (Sigma-Aldrich, Germany) was dried under vacuum at 70 °C for 24 h to remove residual moisture. Afterward, the CMCNF aqueous suspension was gradually added to dried [EMIM]­OAc under continuous stirring. The mixture was heated to 80 °C to facilitate water evaporation and promote homogeneous dispersion of the CMCNF within the ionic liquid matrix. During this process, the abundant hydroxyl and carboxyl groups on the CMCNF surface establish strong hydrogen-bonding and ion–dipole interactions with both the imidazolium cations and acetate anions of the ionic liquid. These interactions enhance interfacial compatibility, suppress macrophase separation, and promote homogeneous confinement of the ionic liquid throughout the nanofibrillar network. After mixing, the system was stirred until a uniform and viscous gel was obtained. The ionogel was subsequently degassed under vacuum to remove entrapped air bubbles. The resulting ionogel was cast into films and dried in a vacuum oven at 70 °C for 12 h to yield mechanically robust ionogel electrolytes. To evaluate the effect of composition on electrochemical and mechanical properties, three representative electrolyte formulations were prepared by varying the CMCNF content while maintaining [EMIM]­OAc as the continuous ion-conducting phase. The samples were designated as IG-5, IG-10, and IG-15, corresponding to CMCNF concentrations of 5, 10, and 15 wt %, respectively. The amount of CMCNF was calculated with respect to the total mass of the precursor mixture prior to casting.

### 2.2 Supercapacitor Assembly

Flexible supercapacitors were assembled in a symmetric sandwich configuration. Electrodes were fabricated using a composite of active carbon YP50F (Kuraray, Kurashiki, Japan), acetylene black (Tianjin Yibo, China) and poly­(vinylidene fluoride) (PVDF, Sigma-Aldrich, Germany) as a binder. These components were thoroughly mixed in a ratio of 80:10:10 wt % to ensure uniform dispersion and optimal electrical conductivity. The resulting slurry was then evenly coated onto a square nickel foam current collector forming the electrodes for the EDLC. For device assembly, the ionogel was employed as both the electrolyte and separator. It was precisely cut to match the dimensions of the electrodes and positioned between two identical electrode layers, creating a symmetrical sandwich structure. Finally, the assembled device was sealed using polyimide tape to prevent electrolyte volatilization and minimize moisture absorption, thereby enhancing the stability and performance of the flexible supercapacitor.

### 2.3 Materials Characterization

The morphology and structure of the synthesized ionogels and hybrid carbon electrodes were investigated using complementary analytical techniques. A field emission scanning electron microscope (Carl Zeiss Merlin FESEM, Oberkochen, Germany) equipped with Energy Dispersive Spectroscopy (EDS) (Oxford Instruments INCA-350 system, United Kingdom) was employed to analyze the microstructure of the ionogel and electrodes. The structural and chemical properties and successful incorporation of the ionic liquid [EMIM]­OAc were evaluated via Fourier-transform infrared spectroscopy (FTIR, Nicolet 6700, Thermo Scientific) equipped with CsI beam splitter and Raman spectroscopy (inVia Qontor, Renishaw). X-ray diffraction (XRD, Bruker, D8-Advance, Billerica, MA, USA) was used to evaluate the crystallinity of the carbon nanomaterials and the amorphous nature of the ionogel. The data collection was carried out at room temperature, between 5° and 60°, with a step size of 0.01° and a collection time of 1 s/step. Phase identification was performed using the JCPDS database and the DIFFRACplus EVA software (V7) by Bruker AXS. To determine the thermal decomposition temperature and solvent content of the electrolyte, thermogravimetric analysis (TGA, Q50 TA Instruments, USA) was performed under a nitrogen atmosphere from room temperature to 600 °C at a heating rate of 10 °C/min. In addition, macroscopic properties such as visual homogeneity, phase stability, and mechanical consistency were qualitatively assessed.

### 2.4 Electrochemical Characterization

The electrochemical performance of the flexible supercapacitors was evaluated in a two-electrode symmetric configuration. Cyclic voltammetry (CV) measurements were conducted within a potential window of 0.0 to 2.0 V at various scan rates ranging from 10 to 100 mV/s to assess the capacitive behavior and rate capability using a bidirectional DC power supply (ITECH IT-M3412, China). Galvanostatic charge–discharge (GCD) profiles were recorded at different current densities (from 0.5 to 10 A/g) to determine the specific capacitance of the devices. Electrochemical Impedance Spectroscopy (EIS) was performed in a frequency range from 100 kHz to 10 mHz with an AC amplitude of 10 mV at open-circuit potential to study the equivalent series resistance (ESR) and ion diffusion kinetics using an AC electrochemical workstation (Metrohm Autolab PGSTAT204, Netherlands). Long-term cycling stability was tested over 10,000 charge–discharge cycles. Additionally, tensile tests on the dog-bone shape ionogel sheet samples at room temperature were performed using a universal testing machine (ZwickRoell, Germany) equipped with a 100 N load cell and pneumatic rubber-coated grips.

## 3 Results and Discussion

### 3.1 Material Characterization

Figure 1a presents the visual appearance of the ionogels at different CMCNF loadings. The optical appearance of ionogels based on [EMIM]­OAc is significantly influenced by the CMCNF content. The sample IG-5 appears highly transparent and nearly colorless, indicating a homogeneous dispersion with minimal light scattering. Upon increasing the CMCNF content (IG-10), the ionogel becomes visibly translucent, exhibiting a slight whitish or pale beige hue, which suggests enhanced light scattering. At 15 wt % CMCNF (IG-15), the material shows a more pronounced opacity and a distinct creamy-yellow coloration. This progressive loss of transparency and shift in color can be attributed to the higher concentration of CMCNF domains, which intensify light scattering and may promote the formation of microscale aggregates, thereby reducing optical clarity.

**Figure 1:**
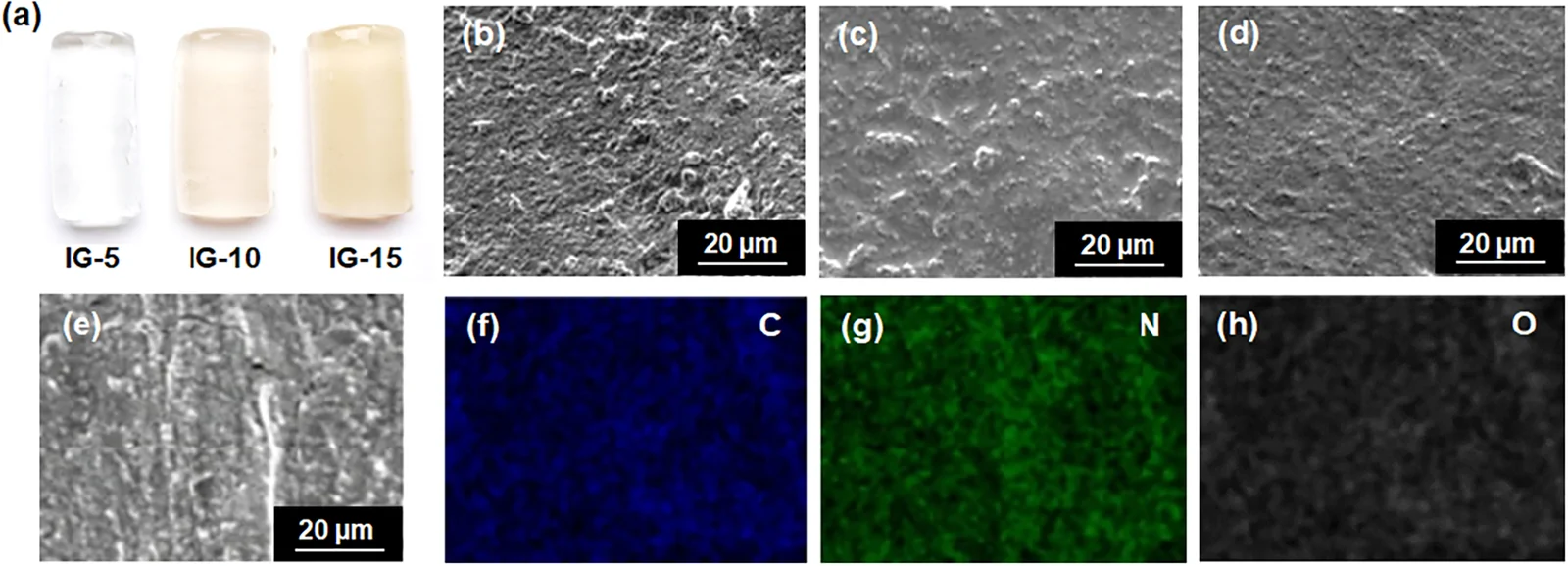
(a) Images of ionogels with different CMCNF contents. FESEM images of: (b) IG-5, (c) IG-10, and (d) IG-15. (e) FESEM image and EDS spectra of: (f) C, (g) N, and (h) O elements for IG-10.

To analyze the microstructural properties of the inogel as a function of the composition in detail, the FESEM micrograph and EDS elemental mappings of the [EMIM]­OAc/CMCNF ionogels have been carried out to provide key insight into its microstructure and compositional homogeneity. In Figure 1b–d, FESEM micrographs of IG-5, IG-10, and IG-15 reveal a homogeneous morphology for all samples, with noticeable differences in surface texture as the CMCNF loading increases. IG-5 exhibits a relatively smooth and less entangled structure, whereas IG-10 and IG-15 display progressively rougher and more interconnected fibrillar networks, indicating improved structural integrity and effective dispersion of CMCNFs in the ionic liquid. The EDS maps confirm a homogeneous distribution of carbon, nitrogen, sulfur, and oxygen across the sample (Figure 1e–h). Carbon arises from both CMCNF and the imidazolium cation, while nitrogen confirms the uniform presence of the [EMIM]+ cation. Oxygen, contributed by both cellulose and the ionic liquid, also appears uniformly dispersed. This overall elemental homogeneity suggests strong interactions between the ionic liquid and CMCNF, likely involving hydrogen bonding and electrostatic forces. Such a well-integrated structure is beneficial for electrochemical applications, as it ensures continuous ion conduction pathways, low internal resistance, and efficient charge transfer. At the same time, the CMCNF network enhances mechanical stability without hindering ionic mobility. Thus, the results confirm the formation of a homogeneous and stable microstructure of the ionogel suitable for high-performance supercapacitor applications.

The FESEM images of the supercapacitor based on the [EMIM]­OAc/CNF ionogel (95:5 wt %) and activated carbon electrodes reveal structural features critical to its performance. In Figure 2a, the cross-sectional image shows a well-defined and symmetric electrode/electrolyte/electrode configuration, with a uniform ionogel layer between both electrodes. The interfaces appear continuous and free of gaps, indicating strong interfacial adhesion, which is essential to minimize resistance and ensure efficient ion transport. The consistent electrolyte thickness also suggests good fabrication control and uniform electric field distribution. At higher magnification (Figure 2b), the micrograph of the ionogel reveals a highly interconnected nanofibrillar network with a densified and completely filled architecture. The fibrils are uniformly distributed throughout the matrix and form a continuous three-dimensional scaffold that successfully embeds and physically confines the ionic liquid within its interstices. No empty voids, obvious macrophase separation, or large ionic-liquid-rich domains are observed, confirming interfacial compatibility between the CMCNF network and [EMIM]­OAc. In Figure 2c, the electrode exhibits a highly porous and interconnected morphology formed by activated carbon. This creates a hierarchical pore structure with micro, meso-, and macropores, where micropores contribute to charge storage and larger pores facilitate ion diffusion. The rough surface further increases the available active sites. This porous network likely allows effective infiltration of the ionogel, maximizing electrode/electrolyte contact and enabling continuous ion transport pathways. Combined with the homogeneous nature of the ionogel, this structure supports low resistance and efficient charge transfer. Thus, the results confirm a well-integrated architecture with strong interfacial contact and hierarchical porosity, favorable for high-performance supercapacitor applications.

**Figure 2:**
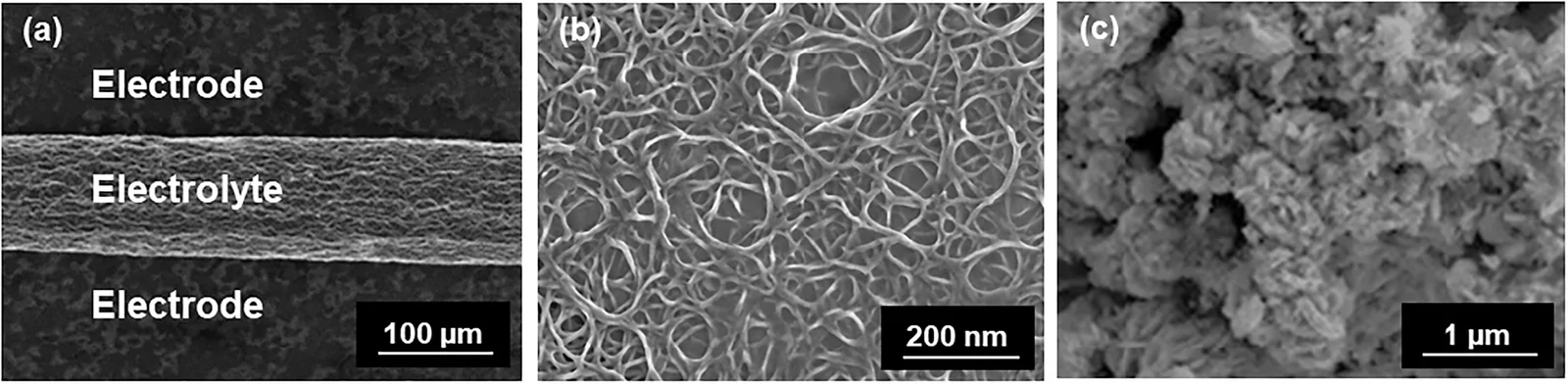
FESEM images of: (a) cross-section of electrode/electrolyte/electrode interfaces, (b) electrolyte and (c) electrode morphology for a supercapacitor with IG-10.

#### 3.1.1 Structural Properties

The molecular interactions within the ionogel framework and its precursors were evaluated via Fourier Transform Infrared (FTIR) and Raman spectroscopy (Figure 3). In the FTIR spectra (Figure 3a), all samples display the characteristic broad O–H stretching band of cellulose in the 3200–3500 cm^–1^ region, which appears slightly shifted and broadened compared to neat CMC, indicating modified hydrogen-bonding environments upon incorporation of the ionic liquid. The bands around ∼2900 cm^–1^ correspond to C–H stretching vibrations, while the region between 1000 and 1200 cm^–1^ is dominated by C–O–C and C–O stretching modes of the polysaccharide backbone. Notably, the appearance and progressive intensification of bands near ∼1560–1600 and ∼1400 cm^–1^ can be attributed to the acetate anion and imidazolium ring vibrations of [EMIM]­OAc, confirming its presence within the ionogel network. Subtle shifts in these bands with increasing ionic liquid content (IG-3 to IG-5) suggest specific intermolecular interactions, likely hydrogen bonding and electrostatic associations, between CMC hydroxyl groups and the ionic liquid ions. Thus, the vibrational spectra confirm the successful formation of CMC-based ionogels and reveal interactions between cellulose nanofibrils and the ionic liquid 1-ethyl-3-methylimidazolium acetate.

**Figure 3:**
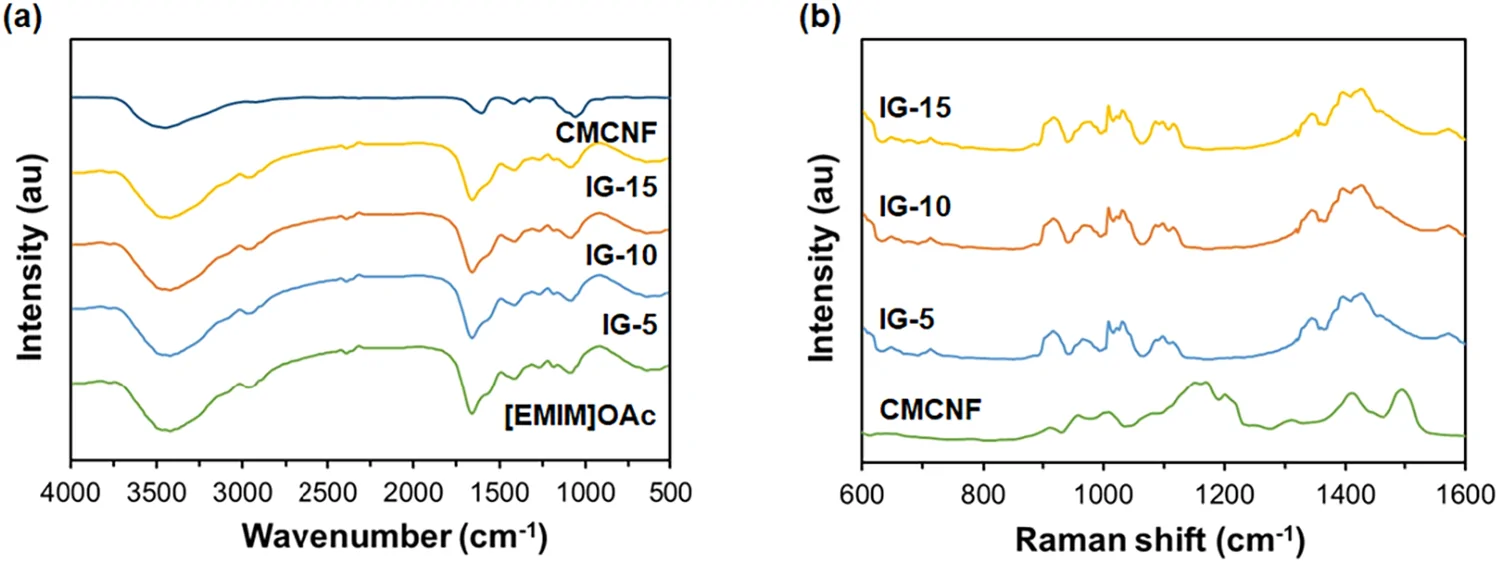
(a) FTIR and (b) Raman spectra of CMCNFc/[EMIM]­OAc ionogels.

The Raman spectra (Figure 3b) further support these findings. The neat CMCNF sample exhibits relatively weak features, whereas ionogel samples show pronounced bands in the 800–1600 cm^–1^ range. The peaks around ∼1000–1100 cm^–1^ are assigned to C–O and C–C stretching modes of cellulose, while the bands near ∼1200–1500 cm^–1^ are associated with imidazolium ring vibrations and acetate-related modes. The increasing intensity and definition of these bands from IG-3 to IG-5 indicate a higher concentration of [EMIM]­OAc and enhanced structural organization within the ionogel matrix. Additionally, slight peak shifts and changes in band shapes suggest strong coupling between CMCNF and the ionic liquid, consistent with the formation of a physically cross-linked network. Thus, both FTIR and Raman analyses demonstrate that the incorporation of [EMIM]­OAc modifies the molecular environment of CMCNF, promoting intermolecular interactions that are essential for ionogel formation and stability.

The structural properties and thermal stability of the ionogels were evaluated by X-ray diffraction (XRD) and thermogravimetric (TG) analysis, confirming the effect of incorporating [EMIM]­OAc into the CMCNF matrix. The XRD patterns (Figure 4a) show that commercial CMC exhibits characteristic diffraction peaks around 2θ ≈ 20–22°, indicative of its semicrystalline structure. In contrast, all ionogel samples display a broad halo centered near 2θ ≈ 20–30°, with a progressive loss of peak sharpness and intensity compared to pristine CMC. This behavior indicates a reduction in crystallinity and an increase in amorphous character upon incorporation of [EMIM]­OAc. The disruption of the native hydrogen-bonding network of CMCNF by the ionic liquid leads to a more disordered structure, consistent with the formation of a homogeneous ionogel. The absence of distinct crystalline peaks from the ionic liquid suggests that it is well dispersed within the polymer matrix rather than phase-separated.

**Figure 4:**
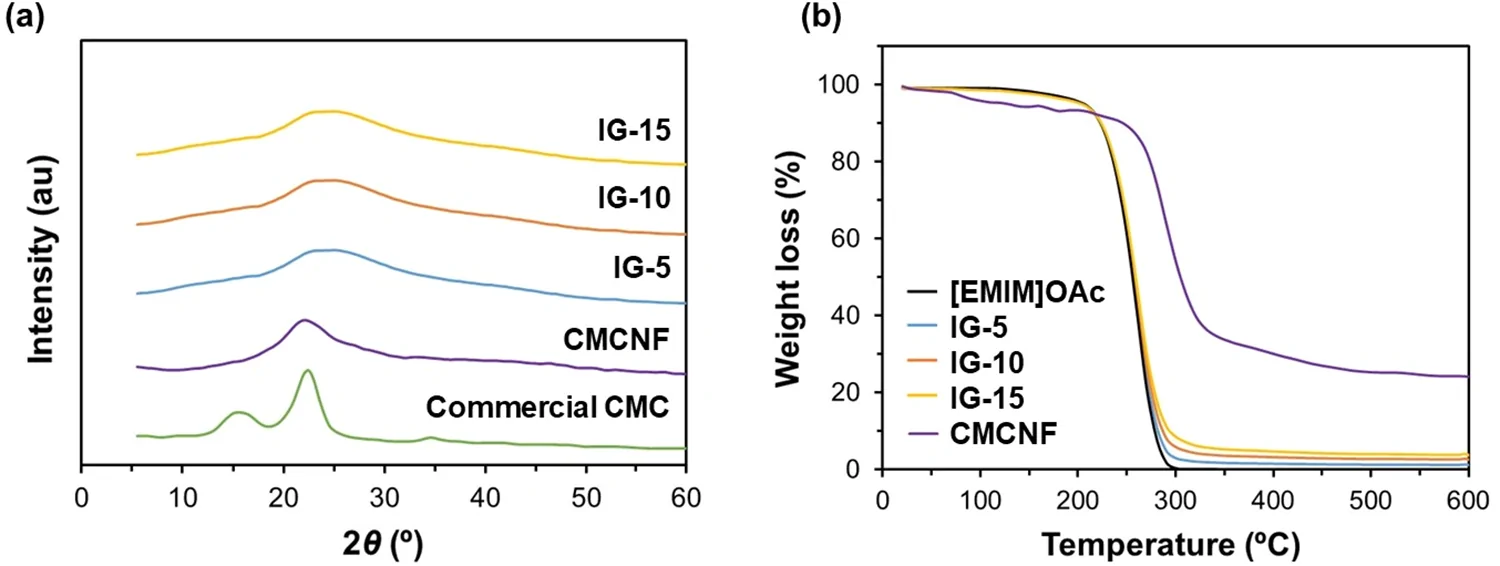
(a) XRD spectra and (b) TGA of [EMIM]­OAc/CMCNF ionogel.

The XRD patterns (Figure 5a) show that commercial CMC exhibits characteristic diffraction peaks around 2θ ≈ 20–22°, indicative of its semicrystalline structure. In contrast, all ionogel samples display a broad halo centered near 2θ ≈ 20–30°, with a progressive loss of peak sharpness and intensity compared to pristine CMC, indicating a marked reduction in crystallinity and the development of a predominantly amorphous structure. This behavior arises from the ability of [EMIM]­OAc to disrupt the intra- and intermolecular hydrogen-bonding network of cellulose, leading to the loss of long-range crystalline order. Notably, the XRD patterns of IG-5 to IG-15 are nearly identical, suggesting that cellulose amorphization is effectively achieved at low ionic liquid contents and that further additions do not induce detectable structural changes, in agreement with previous reports.^69,70^ The absence of distinct crystalline peaks from the ionic liquid further indicates that [EMIM]­OAc is homogeneously dispersed within the polymer matrix rather than phase-separated, supporting the formation of a uniform ionogel.

**Figure 5:**
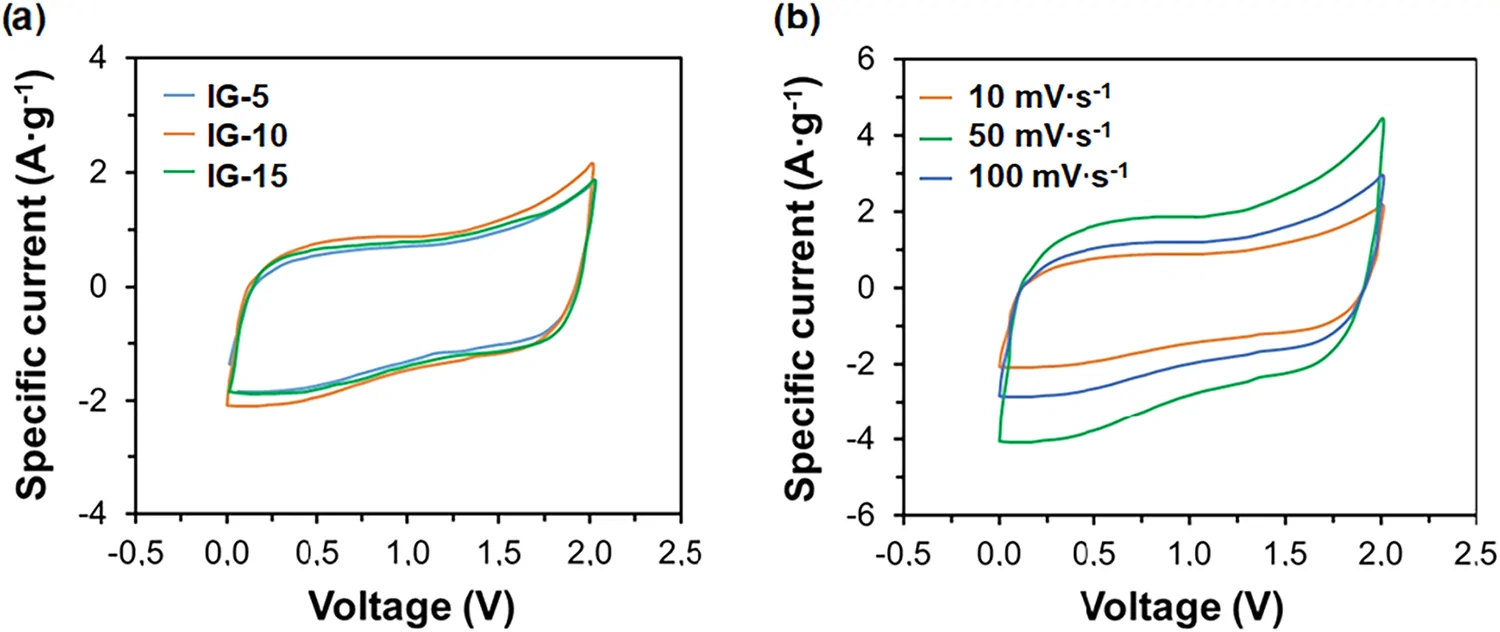
CV (cyclic voltammetry) curves at: (a) 10 mV·s^–1^ scan rates for each [EMIM]­OAc/CNFc ionogel, and (b) for IG-10 at different scan rates.

In Figure 4b, the thermogravimetric profiles show the thermal stability of the neat CMCNF and the [EMIM]­OAc/CNF ionogels. All samples exhibit an initial minor weight loss below ∼120 °C, which can be attributed to the removal of physically adsorbed moisture. Neat CMCNF displays a broad, multistep degradation process starting at ∼250 °C and extending up to ∼400 °C, leaving a relatively high char residue (∼25 wt %) at 600 °C, indicative of its carbonaceous structure. In contrast, the ionogel samples ([EMIM]­OAc incorporated into CMCNF) show a sharper and more pronounced mass loss between ∼240 and ∼300 °C, corresponding to the decomposition of both the cellulose nanofiber network and the ionic liquid. The onset degradation temperature slightly decreases upon incorporation of [EMIM]­OAc, suggesting a plasticizing effect and possible interactions between the ionic liquid and CMCNF that facilitate thermal decomposition. Among the ionogels, IG-15 exhibits a slightly higher residual mass compared to IG-5 and IG-10, which may indicate a higher inorganic or carbonaceous residue or stronger interactions within the network. Therefore, the incorporation of [EMIM]­OAc reduces the thermal stability of CMCNF but leads to distinct decomposition behavior and lower char residues, confirming successful ionogel formation and the influence of ionic liquid content on thermal properties. Thus, the XRD and TGA results demonstrate that the incorporation of [EMIM]­OAc significantly alters the structural order of CMCNF, promoting amorphization, while maintaining adequate thermal stability for ionogel applications.

### 3.2 Electrochemical Performance

#### 3.2.1 Capacitive Behavior and Voltage Window

The electrochemical stability of the [EMIM]­OAc/CMC ionogels was evaluated via Cyclic Voltammetry (CV). Figure 5 presents CV responses of [EMIM]­OAC/CMC ionogels, highlighting both composition-dependent behavior and scan-rate effects. In Figure 5a, all samples exhibit quasi-rectangular CV profiles with slight distortions at higher potentials, indicative of predominantly capacitive behavior with minor resistive contributions. The absence of pronounced redox peaks suggests that charge storage is mainly governed by electric double-layer capacitance rather than faradaic processes. Among the evaluated ionogels, IG-10 displays the highest current response over the entire potential window, pointing to an enhanced specific capacitance compared to IG-5 and IG-15. This improvement can be attributed to an optimal balance between ionic conductivity and electrode/electrolyte interfacial accessibility at intermediate composition. In contrast, IG-5 shows lower current densities, likely due to limited ion transport, while IG-15 presents slightly reduced performance compared to IG-10, possibly as a consequence of increased viscosity or hindered ion mobility at higher ionic liquid content.

Figure 5b shows the CV curves of IG-10 at increasing scan rates (10–100 mV·s^–1^). As the scan rate increases, the current response rises proportionally, while the overall shape of the curves remains largely preserved. This behavior indicates good rate capability and fast charge propagation within the ionogel. However, at higher scan rates, a slight distortion and tilting of the CV profile can be observed, reflecting increased ohmic drop and diffusion limitations. Despite this, the retention of a quasi-rectangular shape even at 100 mV·s^–1^ demonstrates efficient ion transport and low internal resistance.

In summary, the nearly symmetric anodic and cathodic branches confirm good reversibility and high Coulombic efficiency for all ionogels. These results demonstrate that the [EMIM]­OAc/CNFc system, particularly IG-10, is a promising candidate for supercapacitor applications, combining stable electrochemical behavior with efficient charge storage capability.

#### 3.2.2 Charge–Discharge Kinetics and Specific Capacitance

Figure 6 illustrates the galvanostatic charge–discharge (GCD) behavior and rate performance of the supercapacitor based on the IG-10 ionogel. In Figure 6a, the charge–discharge curves recorded at different current densities and 25 °C exhibit nearly symmetric triangular shapes, which is indicative of highly reversible capacitive behavior and efficient charge propagation. The linear voltage–time profiles suggest that the device is predominantly governed by electric double-layer capacitance with minimal faradaic contributions. At lower current density (0.5 A·g^–1^), the discharge time is significantly longer, reflecting higher charge storage capacity. As the current density increases, the discharge time decreases accordingly, which is expected due to reduced ion diffusion time and limited accessibility of active sites at higher rates. A small voltage drop (IR drop) is observed at the discharge curves, becoming more pronounced at higher current densities (e.g., 5 A·g^–1^). This behavior indicates the presence of internal resistance within the device, associated with ionic transport limitations and electrode/electrolyte interface resistance. Nevertheless, the relatively small IR drop and the preservation of the triangular shape even at high current densities demonstrate good conductivity and low internal resistance of the IG-10 ionogel system.

**Figure 6:**
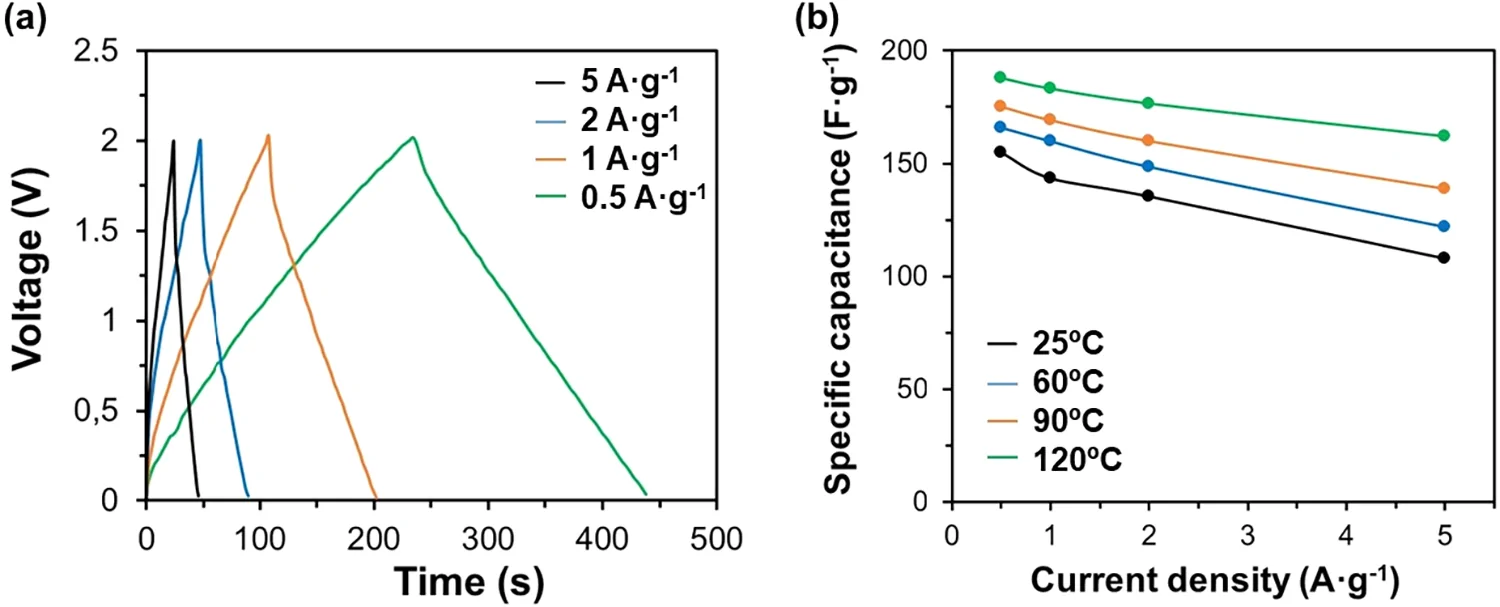
(a) Charge–discharge curves at different current densities at 25 °C, and (b) specific capacitance vs current density for supercapacitor with IG-10.

Figure 6b presents the variation of specific capacitance as a function of current density at different temperatures. At all temperatures, the specific capacitance decreases gradually with increasing current density, which is a typical behavior attributed to insufficient time for electrolyte ions to fully access the porous structure at higher charge–discharge rates. Importantly, a significant enhancement in capacitance is observed with increasing temperature. For instance, the capacitance at 120 °C is consistently higher than that at 25 °C across all current densities. This temperature-dependent improvement can be attributed to enhanced ionic conductivity and reduced viscosity of the ionogel at elevated temperatures, which facilitate faster ion diffusion and better utilization of the electrode surface. Additionally, the relatively moderate decline in capacitance with increasing current density, particularly at higher temperatures, indicates good rate capability and efficient ion transport within the IG-10 system. Thus, the GCD and rate performance results confirm that the IG-10-based supercapacitor combines high capacitance, good reversibility, and strong thermal tolerance, making it a promising candidate for high-temperature energy storage applications.

#### 3.2.3 Impedance Spectroscopy (EIS)

Electrochemical Impedance Spectroscopy (EIS) was performed to further understand the internal resistances. Figure 7 presents the Nyquist plots of the [EMIM]­OAc/CNF ionogels measured at different temperatures. All spectra exhibit a depressed semicircle in the high- to low-frequency region followed by an inclined line at low frequencies, indicating the coexistence of bulk ionic transport and electrode polarization processes. As the temperature increases, the overall impedance decreases significantly, as evidenced by the progressive leftward shift of the curves along the real axis (*Z*′). In particular, the high-frequency intercept, commonly associated with the bulk resistance (*R*_b_), decreases from 25 to 120 °C, confirming the thermally activated nature of ion transport within the ionogel matrix. The reduction in semicircle diameter with increasing temperature further supports the enhancement of ionic conductivity. At low frequencies, the slope of the tail becomes steeper with temperature, suggesting improved ion mobility and reduced diffusion resistance. This behavior can be attributed to increased segmental motion of the CMCNF network and reduced viscosity of the [EMIM]­OAc ionic liquid at high temperatures, facilitating faster charge transport. Thus, the Nyquist response demonstrates that the [EMIM]­OAc/CNF ionogels exhibit improved electrochemical performance at higher temperatures, with reduced resistive and diffusional limitations, highlighting their suitability for applications requiring efficient ionic conduction under thermal conditions.

**Figure 7:**
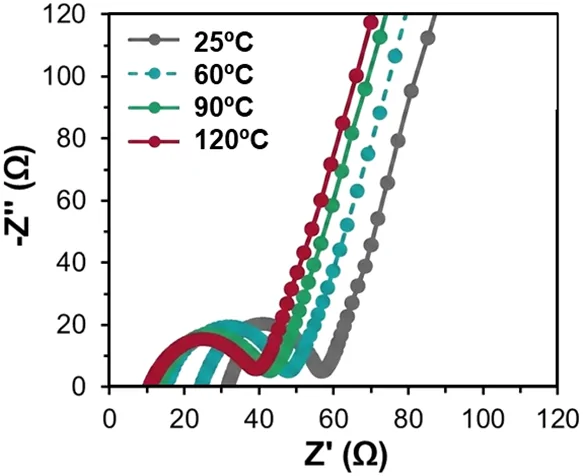
Nyquist plots of [EMIM]­OAc/CNF ionogels at different temperatures.

#### 3.2.4 Mechanical Strength and Cycling Stability

The mechanical performance and electrochemical durability of the supercapacitor with the IG-10 electrolyte was evaluated at room temperature, demonstrating its suitability for flexible energy storage applications. In Figure 8a, the stress–strain curve exhibits a typical nonlinear elastic–plastic behavior. The sample shows an initial linear region at low strain, corresponding to elastic deformation, followed by a gradual increase in stress up to a maximum of approximately 3.5 MPa at a strain close to 90–100%. This indicates good mechanical strength combined with high deformability. The absence of abrupt fracture in the early stage and the relatively large strain at break suggest that the material possesses significant toughness, likely arising from a physically cross-linked network stabilized by hydrogen bonding and ionic interactions within the cellulose-based matrix. The sharp stress drop at the end of the curve corresponds to structural failure, marking the ultimate tensile strength of the device.

**Figure 8:**
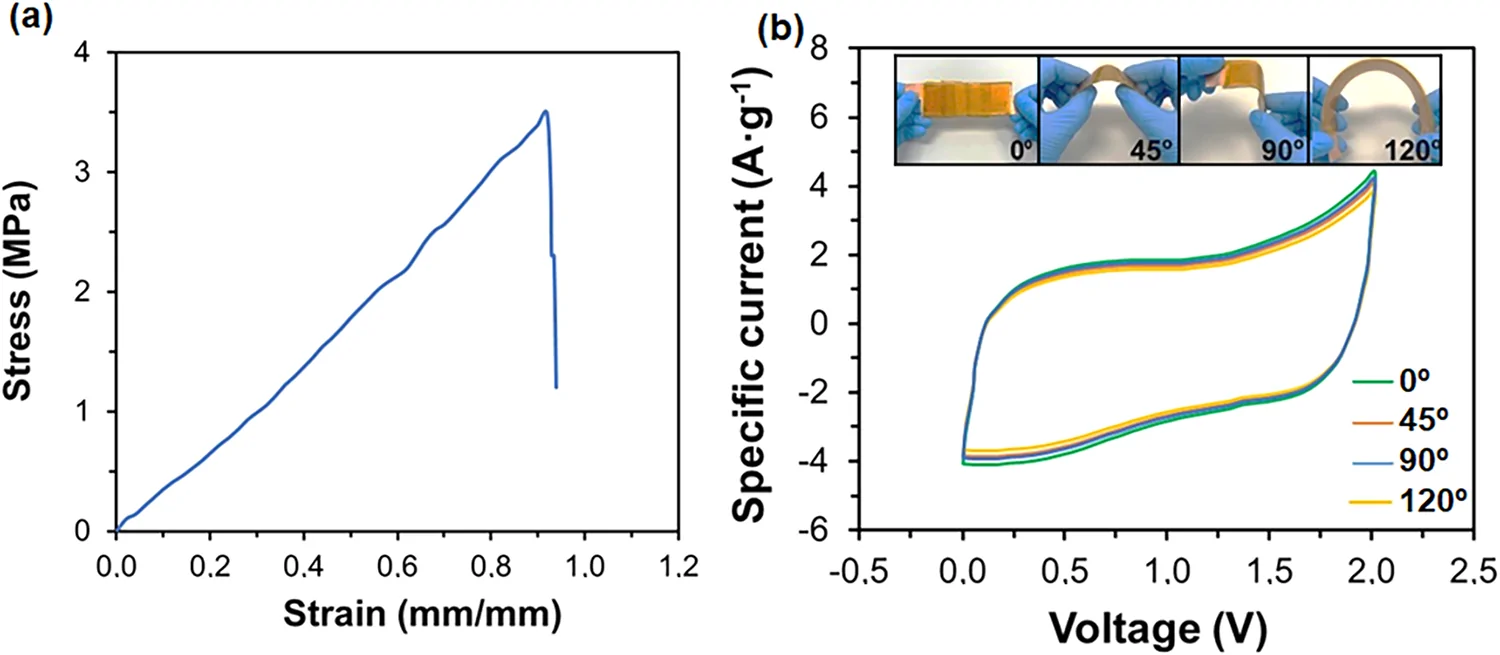
(a) Stress–strain curve, and (b) CV curves of supercapacitor with the IG-10 electrolyte at different bending angles for 50 mV·s^–1^ scan rate.

On the other hand, cyclic voltammetry (CV) curves of the supercapacitor were determined under different bending angles (Figure 8b). The CV profiles maintain a nearly rectangular shape across all deformation states, indicating that the device preserves ideal capacitive behavior and rapid ion/electron transport kinetics. Notably, the curves exhibit a high degree of overlap as the bending angle increases, with only negligible variations in current response. This behavior suggests that the electrochemical performance remains largely unaffected by mechanical deformation, confirming the structural robustness of the electrode/electrolyte interface. Therefore, the results demonstrate remarkable electrochemical stability of the device, highlighting its potential applicability in flexible and wearable energy storage systems.

Furthermore, the cyclic stability results reveal remarkable electrochemical durability of the all-cellulose ionogel electrolyte at 2 A·g^–1^ (Figure 9a). The specific capacitance exhibits a capacitance retention close to 80% after 10,000 charge–discharge cycles. The slight fluctuations observed during the cycling test can be attributed to minor structural rearrangements or interfacial effects; however, no significant performance degradation is evident. This high capacitance retention confirms the stability of the ion transport pathways and the structural integrity of the ionogel under prolonged electrochemical operation. Overall, the combination of favorable mechanical properties and remarkable long-term cycling stability underscores the potential of the all-cellulose ionogel electrolyte for application in flexible and durable electrochemical energy storage devices.

**Figure 9:**
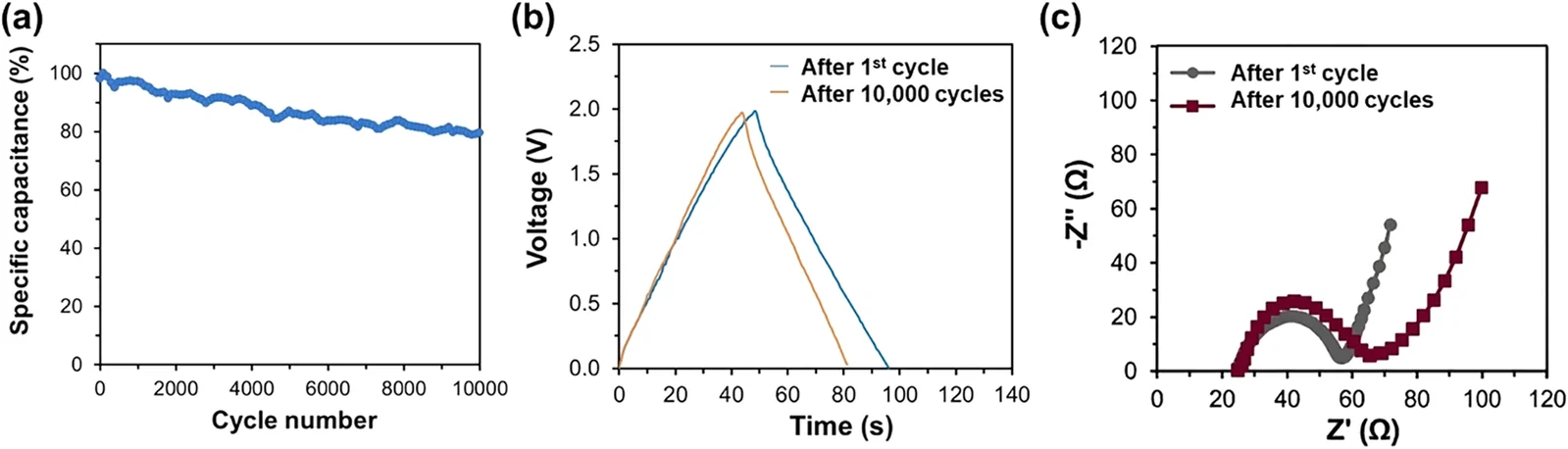
(a) Cyclic stability of all-cellulose ionogel electrolyte at 2 A·g^–1^ for 10,000 cycles at 25 °C, (b) charge–discharge curves at 2 A·g^–1^ at 25 °C, and (c) Nyquist plots for supercapacitor with IG-10 after first cycle and after 10,000 cycles.

To further evaluate the electrochemical performance after long-term cycling, galvanostatic charge–discharge (GCD) profiles were compared after the first cycle and after 10,000 cycles at 2 A·g^–1^ (Figure 9b). The nearly symmetric triangular shapes are well preserved, with only a slight reduction in discharge time after extended cycling. The preservation of the GCD profile indicates highly reversible charge storage behavior and confirms that the ionogel electrolyte maintains efficient ionic transport and low polarization during long-term operation. The small decrease in discharge time is consistent with the minor capacitance loss observed in the cycling test, further supporting the excellent electrochemical stability of the device.

Complementary insights into the long-term stability are provided by electrochemical impedance spectroscopy (EIS). EIS measurements performed after the first cycle and after 10,000 cycles (Figure 9c) provide additional evidence of the stability of the all-cellulose ionogel electrolyte. The Nyquist plots show only moderate changes after extended cycling, with a slight increase in the high-frequency intercept and a slight enlargement of the low-frequency response, indicating a small increase in the equivalent series resistance and charge-transfer resistance. Nevertheless, the overall impedance profile is well preserved, suggesting that the electrode/electrolyte interface remains stable and that ion diffusion pathways are largely maintained throughout prolonged cycling. These EIS results are in good agreement with the high capacitance retention and the nearly unchanged GCD profiles, confirming the outstanding long-term electrochemical durability of the IG-10-based supercapacitor.

#### 3.2.5 Comparative Outlook and Technological Impact

The electrochemical results obtained in this study demonstrate that the CMC–CNF/Silica ionogel-based supercapacitor bridges the gap between high-power aqueous devices and high-energy organic systems. By achieving an operating voltage of 2.0 V, our device offers an energy density significantly higher than commercial supercapacitors using aqueous electrolytes (typically limited to 1.2 V).

Compared to state-of-the-art polymer electrolytes (like PVA-based gels), our cellulose-reinforced ionogel provides superior thermal stability (up to 250 °C), mitigating the risk of thermal runaway and electrolyte evaporation. Furthermore, the use of CMCNF as structural material provides a scalable and cost-effective pathway for the production of green electronics, moving away from hazardous organic solvents and synthetic polymers.

The comparative analysis of CNF-based true ionogel electrolytes (Table 1) shows that the present system, based on CMCNF and [EMIM]­OAc, delivers a specific capacitance of 144 F g^–1^ at 1 A g^–1^, placing it within the typical performance range reported for CNF-based ionogel electrolytes. This value is comparable to CNF/EMIM-based systems (160 F g^–1^,^60^) and CNF-derived polymer ionogels integrated with porous carbon electrodes (153 F g^–1^,^25^), although it is somewhat lower than the upper-end values reported for more structurally complex architectures, such as 174 F g^–1^ in optimized ionogel systems.^74^ It is generally accepted that the specific capacitance in these systems is primarily governed by the electrode architecture rather than the ionogel electrolyte itself. In this context, the obtained value of 144 F g^–1^ still indicates effective ionic transport within the CMCNF-based ionogel and adequate interfacial contact between electrolyte and electrode, without introducing severe diffusion limitations. The observed performance is consistent with other cellulose-derived ionogels, which typically fall within a moderate capacitance window depending on electrode porosity, ionic liquid content, and testing configuration.

**Table 1:** Studies of Supercapacitors Using CNF-Based Ionogel Electrolytes

precursor material (ionogel matrix)	method	specific capacitance (F g^–1^)	reference
CMCNF + EMIM-based IL (EMIMOAc)	CMCNF network used as a scaffold to confine IL, forming a flexible ionogel integrated into solid-state supercapacitors	144 (1 A·g^–1^)	this work
carboxymethylated cellulose CNF + EMIM-based IL (EMIMP)	direct mixing of IL with CNF, and processing into a printable gel polymer electrolyte	160 (1 mA·cm^–2^)	^60^
methyl cellulose (CNF-derived polymer) + EMIM-TFSI IL (95 wt % IL ionogel) + porous CNF electrodes	electrospinning of CNF, KOH activation, and infiltration of IL-rich ionogel	153 (1 A·g^–1^)	^25^
CNF/polysaccharide supramolecular ionogel (glycol chitosan + imidazolium IL)	supramolecular assembly via physical interactions (hydrogen bonding + IL confinement)	40 (1 A·g^–1^)	^71^
CNF–RGO–MoOxNy electrode + IL ionogel electrolyte	freeze-drying (aerogel electrode) and solid-state device with IL-based ionogel	518 (1 A·g^–1^)	^72^
all-cellulose ionogel from low-value wood (CNF-derived matrix) + IL	direct conversion of wood residues into a cellulose network and incorporation of IL	131 (1 A·g^–1^)	^73^
cellulose nanofiber ionogel with IL (flexible solid-state electrolyte system)	CNF network used as a scaffold to confine IL, forming a flexible ionogel integrated into solid-state supercapacitors	174 (1 A·g^–1^)	^74^

From a materials design perspective, the use of CMCNF provides a functionalized nanofibrous scaffold that enhances compatibility with the ionic liquid, promoting homogeneous confinement and maintaining ion mobility within the polymeric network. Although the capacitance is lower than in highly optimized systems, this reflects the trade-off between electrochemical performance and structural simplicity. Indeed, many high-performing literature systems rely on more complex and energy-intensive fabrication routes, such as electrospinning, carbonization, or multistep cross-linking processes.

In summary, the present CMCNF-based ionogel demonstrates a balanced combination of sustainability, simplicity, and electrochemical functionality. While its capacitance is moderate compared to state-of-the-art optimized systems, it remains fully within the performance range of true ionogels reported in the literature, supporting its applicability in flexible and solid-state supercapacitor devices.

## 4 Conclusions

This study demonstrates the successful development of a sustainable and flexible supercapacitor based on a CMCNF-reinforced ionogel electrolyte and flexible carbon electrodes. The proposed ionogel, composed of 1-ethyl-3-methylimidazolium acetate ([EMIM]­OAc) homogeneously distributed within a CMCNF matrix derived from carboxymethyl cellulose (CMC) through a combined physicochemical and mechanical fibrillation process, addresses key limitations associated with conventional aqueous electrolytes, including limited thermal stability and electrolyte leakage. The homogeneous distribution of [EMIM]­OAc throughout the interconnected CMCNF network resulted in an amorphous ionogel structure with enhanced structural stability while facilitating ionic transport.

Furthermore, the assembled supercapacitor delivered a specific capacitance of 144 F g^–1^ at 1 A g^–1^ and operated over an expanded voltage window of 2.0 V, providing higher energy storage capability than conventional aqueous electrolyte systems. Furthermore, the ionogel exhibited significant thermal stability up to 250 °C, overcoming the evaporation and operating temperature limitations commonly observed in aqueous-based devices. The device also provided good mechanical performance, including a tensile strength of 3.5 MPa and flexibility, allowing stable electrochemical performance under different bending conditions.

Overall, these results demonstrate that CMCNF-based ionogels constitute a promising, scalable, cost-effective, and environmentally responsible electrolyte platform for flexible energy-storage devices, combining the mechanical robustness of polymer networks with the wide electrochemical stability window and thermal stability of ionic liquids.
